# Toward the identification of genetic determinants of breast cancer immune responsiveness

**DOI:** 10.1186/2051-1426-3-S1-P1

**Published:** 2015-08-14

**Authors:** Ines Simeone, Wouter Hendrickx, Lance Miller, Halima Bensmail, Ena Wang, Francesco Marincola, Michele Ceccarelli, Davide Bedognetti

**Affiliations:** 1 Qatar Computing Research Institute, Doha, Qatar; 2 Sidra Medical and Research Center, Doha, Qatar; 3 Wake Forest School of Medicine, Winston Salem, NC, USA

## 

Overlapping immune signatures are observed among cancers with a better prognostic connotation and those with an increased likelihood to respond to immunotherapeutic approaches [1,2]. Such signatures qualitatively overlap with those detected during other conditions of immune-mediated tissue destruction such as flares of autoimmunity or allograft rejection [3]. These pathways reflect a process characterized by the coordinated activation of interferon stimulated genes (ISGs), the recruitment of cytotoxic cells through the production of specific chemokine ligands (CXCR3 and CCR5 ligands), and the activation of immune effector function (IEF) genes [4]. We refer to these genes as the Immunologic Constant of Rejection (ICR) [2-4]. Here, we tested up-front the prognostic role of the ICR genes in the TCGA (The Cancer Genome Atlas) breast cancer database. We show that ICR genes can segregate breast cancers in different immune phenotypes characterized by distinctive prognostic connotations. Whether the favorable cancer immune phenotype is driven by the intrinsic genetics of the tumor cells is presently unknown. By mining copy number variation, gene-expression, and exome sequencing data we are currently characterizing breast cancer somatic alterations implicated in the development of this favorable cancer immune phenotype. The results of this analysis will be presented and discussed.
